# Bimodal Function of Anti-TNF Treatment: Shall We Be Concerned about Anti-TNF Treatment in Patients with Rheumatoid Arthritis and Heart Failure?

**DOI:** 10.3390/ijms19061739

**Published:** 2018-06-12

**Authors:** Przemyslaw J. Kotyla

**Affiliations:** Department of Internal Medicine Rheumatology and Clinical Immunology, Medical Faculty in Katowice, Medical University of Silesia, 40-055 Katowice, Poland; pkotyla@sum.edu.pl; Tel.: +48-32-359-8290; Fax: +48-32-202-9933

**Keywords:** tumor necrosis factor, TNF inhibitors, rheumatoid arthritis, heart failure

## Abstract

Treatment with anti-TNF-α (tumor necrosis factor), one of the pivotal cytokines, was introduced to clinical practice at the end of last century and revolutionized the treatment of rheumatoid arthritis (RA) as well as many other inflammatory conditions. Such a treatment may however bring many safety issues regarding infections, tuberculosis, as well as cardiovascular diseases, including heart failure. Given the central role of proinflammatory cytokines in RA, atherosclerosis, and congestive heart failure (CHF), such a treatment might result in better control of the RA process on the one side and improvement of heart function on the other. Unfortunately, at the beginning of this century two randomized controlled trials failed to show any benefit of anti-TNF treatment in patients with heart failure (HF), suggesting direct negative impact of the treatment on morbidity and mortality in HF patients. As a result the anti-TNF treatment is contraindicated in all patients with heart failure and a substantial portion of patients with RA and impaired heart function are not able to benefit from the treatment. The role of TNF in CHF and RA differs substantially with regard to the source and pathophysiological function of the cytokine in both conditions, therefore negative data from CHF studies should be interpreted with caution. At least some of RA patients with heart failure may benefit from anti-TNF treatment, as it results not only in the reduction of inflammation but also contributes significantly to the improvement of cardiac function. The paper addresses the epidemiological data of safety of anti-TNF treatment in RA patients with the special emphasis to basic pathophysiological mechanisms via which TNF may act differently in both diseases.

## 1. Introduction

Rheumatoid arthritis (RA) is a chronic, devastating polyarthropathy with symmetrical involvement of peripheral joints [1]. Having a prevalence of 1%, RA is recognized as the most common form of inflammatory polyarthropathy [2]. Inflammation in joints contributes to cartilage damage with formation of bone erosions followed by joint space narrowing. The disease leads to disability, particularly if poorly controlled and is also a leading cause of premature death. The etiology of the disease although not fully understood comprises a variety of factors including environmental, genetic and lifestyle, and sex related [3,4]. Quite recently a dysregulated microbiome as a pathogenic factor in the development of autoimmune disorders, including rheumatoid arthritis has been extensively debated that provided a new insight the on role of bacterial composition on autoimmune response [5].

The inflammatory process starts with breaking the tolerance of immunocompetent cells—mainly T and B cells against self-antigen (antigens). This ultimately leads to an uncontrolled immune response [6]. Recent advances in the understanding of pathogenesis highlighted the role of the cytokine network in the initiation and progression of the disease as well as autoimmune follicular Th (Tfh) response [7,8,9]. The systemic character of the disease brings many pathophysiological consequences and the chronic inflammatory process contributes to such comorbidities as premature atherosclerosis, lipid profile abnormalities, insulin resistance, malignancy, infections, and congestive heart failure (CHF) [10,11,12,13]. The role of systemic inflammation in the development of cardiovascular disease has been continuously addressed in the recent literature. It postulates inflammation as a common trunk for the development of cardiovascular events and inflammatory diseases including RA, systemic lupus erythematosus, and psoriatic arthritis. This may at least partially explain why individuals with inflammatory diseases may be prone to developing severe cardiovascular complications, leading ultimately to premature death for cardiovascular diseases. Indeed, in addition to disability, RA is associated with increased mortality due to cardiovascular disease.

Patients with RA have 1.5 to 2.0 fold increased risk for the development of cardiovascular events and an increased risk of up to 50% compared with the general population [14,15,16,17]. Recent data from a Canadian RA registry showed increased mortality for all-cause and specific causes in RA relative to the general population. The study highlighted the high excess mortality among RA patients under 45 years of age for respiratory and circulatory diseases [18]. The same conclusions came from a recent Danish study where rheumatoid arthritis patients had higher rates of HF and other cardiovascular diseases and RA was associated with a higher risk of developing CHF [19].

The mechanisms leading to increased susceptibility are not fully understood, but growing evidence suggests that it may be the result of the coexistence of traditional risk factors and enhanced inflammation [20,21,22]. In the majority of cases, patients with RA display an unusual presentation of the disease that does not attract sufficient attention of their physicians. Additionally, increased risk for development of cardiovascular complication may be present many years before the onset of RA and its formal diagnosis [23]. As a result, individuals suffering from RA are more than three times more likely to have had myocardial infarction before the diagnosis of RA than people free of disease [24]. Patients with RA are also at higher risk for the development of heart failure [25]. This is seen especially in sero-positive subjects (patients characterized by the presence of rheumatoid factor or antibodies against citrullinated peptides in their sera) [26]. High activity of RA may superimpose symptoms of heart insufficiency, and CHF symptoms may be wrongly attributed to RA activity (e.g. fatigue, general weakness, and a reduction of daily activity). As a result, patients with RA and heart failure are diagnosed later, have less aggressive treatment, and a poorer outcome. The suggested mechanisms at least partially responsible for cardiovascular morbidity and mortality cover direct and indirect cytokine influence, accelerated atherosclerosis and disease-related reduced physical activity [27,28,29]. 

Synthetic disease modifying anti rheumatic drugs (DMARDs) (mainly methotrexate) are still recognized as anchor drugs for the treatment for RA. Unfortunately, only some of the patients respond for such a treatment and there is still a need for more aggressive treatment for the patients. This led to the development of a novel class of drugs for rheumatoid arthritis directly targeting cytokines, co-stimulatory molecules or causing depletion of whole lines of immune cells [30]. This new class of drugs called biologics or biological DMARDs (bDMARDs) revolutionized treatment of RA [31,32,33]. Among biologics targeting cytokines, tumor necrosis factor (TNF)-inhibitors, the first class of biologics commonly introduced to clinical practice still have a slight preference over other biologics due to the availability of long-term safety data, and reasonable cost-effectiveness [34]. This kind of treatment has, however, some limitations as not all patients are suitable for such treatment. Moreover, in the course of treatment some patients may experience adverse drug reactions with tuberculosis, infections, malignancy, neuropathy, and cardiovascular complications being the most frequent reported ones [35,36,37,38,39,40].

Treatment with anti-TNF-α, one of the pivotal cytokine, that has been introduced to clinical practice at the end of last century revolutionized treatment of RA. Given the central role of proinflammatory cytokines in RA, atherosclerosis, and congestive heart failure such a treatment might result in better control of both the RA process on the one side and improvement of the heart function on the other [41,42,43,44]. It is, however, not clear if this benefit exists and whether it is due to the effective suppression of inflammation or targeting TNF, implicated in CHD. 

## 2. The Role of TNF in Physiological and Pathological Conditions

TNF is a classic pleiotropic cytokine and is recognized as one of the key cytokines of inflammation in non-malignant and malignant diseases [45,46,47]. The main sources of this pro-inflammatory cytokine are macrophages and neutrophils, but in special circumstances it can also be produced by T and B lymphocytes and NK cells [48,49,50]. The other source that plays an important role in TNF synthesis are non-immune cells such as endothelial cells, mast cells, smooth and cardiac muscle cells, fibroblasts, and osteoclasts [51]. TNF is synthetized as a transmembrane-bound protein. After cleavage with tumor necrosis factor converting enzyme (TACE), the soluble form of the cytokine is released and may interact with two types of receptors TNFR1 (also sometimes referred to a p55/p60 Cd120a) and TNFR2 (as known as p75/p80 Cd120b) [52,53]. TNF receptors may exist in soluble or membrane bound forms. TNFR1 has similar affinity to soluble and membrane bound forms of TNF since membrane TNF bounds preferentially to TNFR2 [54,55]. Pathophysiological functions of TNFR1 and TNFR2 differs significantly. TNFR1 is linked to the so-called death domain (DD), which effectively activates apoptotic and necroptotic pathways resulting in cell death [56,57,58,59]. TNFR1 is detected on nearly all kinds of cells and predominantly sequestered in the Golgi apparatus [60]. Contrary to this, TNFR2, which lacks the death domain, exhibits far more limited expression and is typically localized in T-lymphocytes, cardiomyocytes, human mesenchymal stem cells, human and murine cardiac resident stem cells, nervous system cells, and thymocytes [61,62,63]. Most biological responses are due to the activation of TNFR1, but some responses have been attributed to the activation of TNFR2. The role of the signal transduction through TNFR1 has been determined in the last years, whereas the signaling through TNFR2 is still awaiting to be explained satisfactorily, although recent data suggests that it plays an essential role in cell proliferation, survival and in the activation of regulatory T cells [64,65].Taking the above into consideration it is clear that the final effect of the cytokine is dependent on the type of receptor it activates, so that one cytokine may exert different physiological effects.

## 3. The Role of TNF in Heart Failure 

The role of TNF in congestive heart failure has been commonly accepted [66,67]. Elevated levels of TNF are seen in CHF patients and it is strongly correlated with the NYHA scale [68]. In the failing heart, TNF contributes to the contractile dysfunction, provokes heart hypertrophy, and induces apoptosis of cardiac myocytes [69]. Several potential mechanisms may be responsible for this process with beta adrenergic receptors uncoupling, oxygen species formation, and the activation of inducible nitric oxygen synthase being the most often observed ones [70,71,72,73,74]. Moreover, chronic stimulation with TNF increases the formation of other proinflammatory cytokines such as IL-6 and IL-1 that are also involved in the pathogenesis of CHF [75,76]. This is an important observation, since the combination of these cytokines exerts a more profound cardiodepressive effect than either of them alone. Loss of function of beta receptors is recognized as the most important mechanism involved in the pathogenesis of CHF. Chronic stimulation of beta adrenoreceptors (β-AR) contributes to increased synthesis of proinflammatory cytokines [77]. As an example, in a rat model, chronic low-dose infusion of the β-AR agonist isoproterenol (ISO) induces load-independent left ventricular hypertrophy and oxidative stress despite the absence of overt myocardial toxicity [78]. In this model β-AR activation also induces inflammatory responses in the heart; ISO infusion in vivo activates nuclear factor-κB (NF)-κB, IL-1β and IL-6 in the heart [79,80] Chronic over-stimulation with TNF resulted in impaired ability of the β-AR to respond to natural agonists. This occurred without the reduction of receptor density and binding affinity. Desensitization of β-AR after exposure to the high amounts of TNF is mediated via the interaction of TNF and GPCR kinases, which in turn phosphorylate β-AR and reduce their activity. In line with this β-AR are not only a victim of TNF over-expression but also act in propagation of the inflammatory state. In some pathophysiological conditions TNF may, however play the protective role, limiting infarct size and provide immunoregulatory activities in states of heart dysfunction [81,82]. It should be noted that low levels of tissue TNF seem to be necessary for the protection of the myocardium against injury, while higher systemic TNF contributes to the development of ventricular dysfunction [83]. Experimental studies with mammalian cardiomyocytes showed that TNF exerts a direct inotropic negative effect, reduces viability of cardiomyocytes, enhances myocardial fibrosis and ventricular hypertrophy. Overexpression of TNF leads to a reduced response to beta adrenergic stimulation and heart remodeling. Given the pivotal role of TNF in pathogenesis of CHF, it was speculated that inhibition of the cytokine may bring therapeutic effect in patients with CHF. This was the theoretical background for two multicenter clinical trials RENAISSANCE (Randomized Etanercept North American Strategy to study antagonism of cytokines) and RECOVER (research into Etanercept cytokine antagonism in vascular dysfunction) that examined the utility of TNF inhibition with Etanercept (ETA). Those studies have similar designs but differ with regard to the dose of ETA used with 25 mg ETA given twice a week versus 25 mg three times per week and 25 mg once a week versus 25 mg twice a week for RENAISSANCE and RECOVER, respectively. The results from both studies were summarized in the RENEWAL (the randomized etanercept worldwide evaluation) study that focused on all-cause mortality and hospitalization for heart failure [84]. The studies failed to show any benefit with the treatment of CHF with etanercept. The ATTACH (anti-TNF therapy against congestive heart failure) study, that investigated infliximab (INF) in dose 5 versus 10 mg/kg in patients with moderate to severe heart failure, also failed to show any improvement in heart function [85]. Moreover, the higher dose of INF contributed to the worsening of heart failure and the reduction of lifespan. The results and subsequent limitation driven from CHF studies were translated directly to all indications for anti-TNF therapy including RA, inflammatory bowel diseases, psoriatic arthritis and spondyloarthropathies. As a result, patients with impaired heart function are not usually recommended to be given anti-TNF treatment. 

## 4. Types of Heart Failure

Congestive heart failure (CHF) is one of the most common disorders in current times. The prevalence of diseases still rising, partially due to improvement in the treatment of acute coronary states and improvement of the treatment of the other cardiovascular diseases; however, not all causes of CHF are the same. Based on the clinical judgement and the results of assessment of the heart function (mainly by echocardiography) two distinctive types of heart failures (HF) may be distinguished: HF with preserved left ventricular function (heart failure with preserved ejection fraction- HFpEF; EF > 50%) and HF where left ventricular function is significantly compromised (heart failure with reduced ejection fraction-HFrEF; EF < 50%) [86]. HFpEF usually develops as the results of low grade inflammation commonly presented in diabetes, chronic obstructive pulmonary disease, and obesity, while HFrEF is the result of acute heart injuries as coronary heart disease, uncontrolled hypertension or myocardial infarction (MI) [87]. The heart structure and type of dysfunction vary across two types of HF. For HFpEF, structure of the heart is generally preserved with only concentric remodeling seen, while HFrEF is characterized by deep changes in heart structure with marked eccentric remodeling, heart dilatation, and subsequent systolic insufficiency [88,89]. In the last decade a novel pathophysiological mechanism for CHF has been proposed, based on the role of inflammation in genesis and propagation of the disease. Inflammation and activity of proinflammatory cytokines play the role in both forms, however to a different extent. With respect to HFpEF, low grade inflammation is responsible for endothelial inflammation and damage, activation of heart myofibroblasts to synthetize inflammatory cytokines, expression of cytokines, and receptor chemokines, thereby facilitating infiltration of the heart with immune cells. In line with this, Mantel et al showed increased risk for HF in patients with RA who were free from ischemic heart disease raising the suggestion that for the RA population systemic inflammation rather than structural damage of the heart plays a key role [90]. Similarly to RA, in advanced HFrEF in a RA-free population endothelial dysfunction is also present and attributed to high levels of proinflammatory cytokines [91]. Unlike RA, the increased plasma levels of cytokines (IL-6 and TNF) in HFrEF patients do not result from systemic inflammation but result from severity of HFrEF [91]. Contrary to the general population, where HF is usually the result of ischemic heart disease (IHD) or previous MI, most patients with RA suffer from HFpEF [92,93], that is attributable to systemic inflammation and endothelial dysfunction [94]. As a result we face two distinct diseases; HF in general population and HF in RA patients with striking differences regarding the pathophysiology, heart structure, function, and finally the role of proinflammatory cytokines including TNF.

So, the open question remains: if patients with CHF and RA are the same, does the restriction from the treatment of one condition limit the treatment of the other disease?

## 5. The Role of TNF in Rheumatoid Arthritis and Heart Failure

All pathophysiological states where inflammation plays a role are similar with regards to high levels of proinflammatory cytokines and recruitment of immunocompetent cells. These processes are seen in such diseases as diabetes, sepsis, connective tissue diseases, inflammatory arthropaties, atherosclerosis, and heart failure. The striking similarity is elevated levels of TNF in CHF and RA, as in both TNF levels correlate with disease activity (disease activity score-DAS and New York Heart Asscociation-NYHA respectively). Patients with CHF and RA are characterized by comparable levels of TNF in serum [95,96,97,98]. In line with this pathophysiological action, TNF may not differ between two diseases. It is however not entirely clear whether TNF plays a causative role in both conditions. In RA, TNF together with other proinflammatory cytokines orchestrates the inflammatory response, thus cytokine inhibition contributes to a reduction of disease activity in a substantial proportion of the patients. In CHF patients, the effect of TNF may not be so clear and probably depends on the source of TNF. The source of TNF in CHF is still a matter of debate and there are several hypotheses pointing to different sources of TNF in various forms of heart failure. The first hypothesis suggested that in CHF, the heart alone is the main source of TNF although this depends on the stage of insufficiency [99]. The second hypothesis indicates extra-cardiac tissues as the source of TNF, which is produced as part of systemic low grade inflammation as seen in obesity, diabetes, and hypertension [100]. The other theory claimed that TNF is the one arm of immune response and is synthesized mainly by several peripheral tissues involved in immune response (for RA it would be synovial tissues and immunocompetent cells). Peripheral tissues may also produce high amounts of TNF as the result of oxidative stress and generalized hypoxia. In rheumatoid arthritis, where TNF is synthetized in extra-cardiac tissues, a high level of cytokine is also linked with cardiovascular morbidity. Synovial membranes express high concentrations of proinflammatory cytokines including TNF [101]. The other source of TNF in RA is interferon-γ (IFN-γ) stimulated macrophages that infiltrate the synovium followed by the production of large amounts of TNF, eventually causing joint destruction, but also acting more systemically and influencing heart function. Therefore, in spite of the similar concentration of TNF in CHF and RA there are striking dissimilarities between both conditions that may explain various TNF function in both diseases. The source and probably the function of TNF differs between the two diseases. In CHF, TNF synthetized by the failing heart may in some extent play a compensative role that may stabilize heart function. A key phenomena existing is the two types of TNF receptors playing diverse roles in myocardial homeostasis [102]. TNFR1 and TNFR2 are independently regulated and mediate different cardiac responses, so that effects of TNF stimulation will depend on TNFR expression patterns. Signaling through TNFR1 appears detrimental and may explain the cardiotoxic effect of TNF, while TNFR2 appears to mediate cardioprotective effects [103,104]. Indeed, lessons derived from animal models showed that chronic hypoxia contributes to the high expression of TNF in the heart of rats with concomitant increased expression of TNFR2 and this response was abolished by infliximab [105]. In human cardiac tissues under ischemic injury local production of TNF coupled with hypoxia-mediated induction of TNFR2 facilitate the transient proliferation and eventually differentiation of resident cardiac stem cells into mature cardiac myocytes [106]. Contrary to this in various models of experimental cardiac hypertrophy, TNF inhibitor was able to reduce heart hypertrophy, which was believed to be mediated by a reduction of levels of myocardial hypertrophy marker genes, including atrial natriuretic factor (ANF), matrix metalloproteinase (MMP)-9, and MMP-13 [107]. It is plausible that in these models TNF inhibitors blocks TNF signaling mediated by TNFR1 [108]. Moreover, as it was shown in patients with heart failure, patients with HFpEF are characterized by higher TNFR2 levels when compared with HFrEF patients, which suggests a direct cardioprotective function of TNFR2 signaling in humans [109].

Thus, the function of TNF depends on the type of receptor preferentially activated by the cytokine that is mainly dependent on the concentration of the cytokine. Moreover, it is well established that in states of low grade inflammation (where TNF is produced as the part of systemic inflammation) the reduction of inflammatory process contributes significantly to the improvement of heart function. Finally, it does not seem to be reasonable to transfer negative data from one disease to the other, even when both diseases are characterized by high levels of the same cytokine.

When we extract data from the ATTACH study we may find that after every infusion of infliximab the level of TNF has risen significantly, reaching values that extended many times the concentrations observed before the first infusion [85]. In the ATTACH study, biologically active TNF was not detected in serum samples with elevated levels of immunoreactive TNF, which brought suspicion that the assay used for the measurement of the serum TNF detected not only free TNF but also TNF–infliximab complexes, which were immunoreactive but biologically inactive. Even if this is true, the source of this high amount of TNF remains obscure. Quite recently we and others observed a similar elevation of TNF in a study with etanercept, which indicates that elevation of TNF after anti-TNF treatment is a common phenomenon [97,110,111,112]. Increase of TNF after anti-TNF therapy, however, was not reported in all studies [113]. Elevation of TNF may therefore explain the cardiotoxic effect of anti-TNF agents, however it is not clear whether this cardiotoxicity is clinically relevant to all conditions where increment of TNF is observed or is restricted only to patients with heart failure. Another possible mechanism that may be at least partially responsible for the cardiotoxic effects of anti-TNF agents is the “reverse signaling [114,115]”. TNF expressed on cardiomyocytes of the failing heart may behave as receptors, activating intracellular signaling pathways when interacting with TNF antagonists, thus acting to potentiate the toxic effect of the cytokine. In RA where TNF is expressed mainly in synoviocytes but not in cardiomyocytes this effect probably does not play a role. This may explain the different actions of TNF inhibition in both diseases. This speculation however waits to be determined.

## 6. Anti-TNF Treatment in a Real World Clinic

Recently Al-Aly et al. undertook an analysis of the effect of TNF blockade on the risk of cardiovascular outcomes in patients with RA. In the study, TNF administration was not associated with increased risk of cardiovascular events regardless of previous heart disease. Moreover, TNF treatment was associated with a decreased risk of cardiovascular events that was seen in a younger subgroups of patients. All group analysis failed to show any significant change in the risk of cardiovascular events in any other subgroup [116].

In line with this, data derived from a RA registry showed that heart failure is less common in TNF inhibitor exposed patients with RA when compared with no TNF exposure RA patients [117]. This observation was substantiated by the large study of Listing et al. [118]. The data from the German Biologic Register indicates that TNF inhibitor treatment is more likely to be beneficial than harmful with regard to the risk of heart failure and this protective effect is due to reduction of the inflammatory activity of RA. Furthermore, the data suggest that treatment with anti-TNF does not increase the risk of worsening of prevalent heart failure [118]. Indeed, recently Arts et al. showed that the suppression of inflammation and reduction of disease activity exert a protective effect on the cardiovascular system. In the study, low disease activity measured by disease activity score (DAS28) was significantly associated with a reduced risk of coronary vascular disease [119]. While observational studies give an insight into the risk of CHF development and incidence of cardiovascular events, the weak point of these studies is the fact that they cannot directly answer the question: Is it safe to use anti-TNF treatment in RA patients regardless of their heart function? The only proper way to definitively answer this question is by using a randomized controlled clinical trial design [120]. As the anti-TNFs are contraindicated in treatment of patients with compromised heart function these studies may bring many bioethical issues. In our small study where we tried to assess heart function in a group of patients treated with infliximab, we actually showed that such a treatment contributes to the improvement of heart function and a reduction of the biomarkers of heart failure. However, the weak point of this study was that recruitment was only allowed for patients with preserved left ventricular function, which limits our ability to draw final conclusions [121]. In a similar study in patients with RA and without cardiovascular diseases Vizzardi et al. showed that one year of treatment with anti-TNF did not affect myocardial contractility, proving a neutral activity of TNF antagonist on normal left heart function [122]. Unfortunately, this study again cannot answer the question how TNF antagonists work in CHF patients with RA.

## 7. Conclusions

At the moment, we are still at the crossroads trying to help all RA patients and provide them with modern effective treatment. A substantial proportion of them may have impaired heart function as the inflammatory process alone is a strong causative factor for the development of heart failure. Many facts suggest that the suppression of inflammation may bring a cardioprotective effect in RA patients. But it is still unclear whether TNF acts in the same way in CHF and RA patients. Fortunately, development of new biologic with different mode of action; e.g., IL-6 antagonists, B cell depletion therapy or the introduction of JAK kinase inhibitors may offer a therapeutic option when TNF therapy is contraindicated. 

Take Home Messages:

1. Heart failure in the general population and in inflammatory conditions vary with regard to the potential mechanisms and the source of inflammatory cytokines.
2. Contrary to heart failure in the general population where TNF is produced by the failing heart and in some extent may play a cardioprotective role, in RA patients it is synthesized by synoviocytes and immunocompetent cells and may exert a detrimental effect on heart function.
3. TNF blockade in RA patients suppresses the inflammatory response and directly translates to the improvement of heart function.
4. Direct translation of negative data in HF patients treated with anti-TNF and should be done with caution as it is not possible to compare two different diseases and the doses of anti-TNF agents in HF studies were 2–3 times higher than those used for the treatment of RA
5. It is plausible that TNF inhibition brings different pathophysiological consequences in HF and RA patients as the result of crosstalk between TNF inflammatory cytokines, adipokines and apoptotic, and necroptotic signaling molecules.
